# Comparison of the diagnostic accuracy of monocyte distribution width and procalcitonin in sepsis cases in the emergency department: a prospective cohort study

**DOI:** 10.1186/s12879-021-06999-4

**Published:** 2022-01-04

**Authors:** Chih-Huang Li, Chen-June Seak, Chung-Hsien Chaou, Tse‐Hsuan Su, Shi-Ying Gao, Cheng-Yu Chien, Chip-Jin Ng

**Affiliations:** 1 Department of Emergency Medicine, Linkou Medical Center, Chang-Gung Memorial Hospital, No. 5, Fuxing St., Guishan Dist., Taoyuan City 333, Taiwan (R.O.C.); 2 Graduate Institute of Clinical Medical Sciences, Chang-Gung University, Taoyuan, Taiwan; 3 College of Medicine, Chang-Gung University, Taoyuan, Taiwan; 4 Chang-Gung Medical Education Research Centre, Chang-Gung Memorial Hospital, Taoyuan, Taiwan; 5 Department of Emergency Medicine, Ton-Yen General Hospital, Zhubei, Taiwan

**Keywords:** Monocyte, Sepsis, Biomarker, Diagnosis, Emergency department

## Abstract

**Background:**

Early diagnosis and treatment of patients with sepsis reduce mortality significantly. In terms of exploring new diagnostic tools of sepsis, monocyte distribution width (MDW), as part of the white blood cell (WBC) differential count, was first reported in 2017. MDW greater than 20 and abnormal WBC count together provided a satisfactory accuracy and was proposed as a novel diagnostic tool of sepsis. This study aimed to compare MDW and procalcitonin (PCT)’s diagnostic accuracy on sepsis in the emergency department.

**Methods:**

This was a single-center prospective cohort study. Laboratory examinations including complete blood cell and differentiation count (CBC/DC), MDW, PCT were obtained while arriving at the ED. We divided patients into non-infection, infection without systemic inflammatory response syndrome (SIRS), infection with SIRS, and sepsis-3 groups. This study’s primary outcome is the sensitivity and specificity of MDW, PCT, and MDW + WBC in differentiating septic and non-septic patients. In addition, the cut-off value for MDW was established to maximize sensitivity at an optimal level of specificity.

**Results:**

From May 2019 to September 2020, 402 patients were enrolled for data analysis. Patient number in each group was: non-infection 64 (15.9%), infection without SIRS 82 (20.4%), infection with SIRS 202 (50.2%), sepsis-3 15 (7.6%). The AUC of MDW, PCT, and MDW + WBC to predict infection with SIRS was 0.753, 0.704, and 0.784, respectively (p < 0.01). The sensitivity, specificity, PPV, and NPV of MDW using 20 as the cutoff were 86.4%, 54.2%, 76.4%, and 70%, compared to 32.9%, 88%, 82.5%, and 43.4% using 0.5 ng/mL as the PCT cutoff value. On combing MDW and WBC count, the sensitivity and NPV further increased to 93.4% and 80.3%, respectively. In terms of predicting sepsis-3, the AUC of MDW, PCT, and MDW + WBC was 0.72, 0.73, and 0.70, respectively. MDW, using 20 as cutoff, exhibited sensitivity, specificity, PPV, and NPV of 90.6%, 37.1%, 18.7%, and 96.1%, respectively, compared to 49.1%, 78.6%, 26.8%, and 90.6% when 0.5 ng/mL PCT was used as cutoff.

**Conclusions:**

In conclusion, MDW is a more sensitive biomarker than PCT in predicting infection-related SIRS and sepsis-3 in the ED. MDW < 20 shows a higher NPV to exclude sepsis-3. Combining MDW and WBC count further improves the accuracy in predicting infection with SIRS but not sepsis-3.

*Trial registration* The study was retrospectively registered to the ClinicalTrial.gov (NCT04322942) on March 26th, 2020.

**Supplementary Information:**

The online version contains supplementary material available at 10.1186/s12879-021-06999-4.

## Background

Sepsis is a life-threatening organ dysfunction caused by a dysregulated host response to infection [1–3]. The average mortality due to sepsis is about 30 to 40% [4, 5]. Despite the new Sepsis-3 definition that focuses mainly on patients with the worst outcome, early recognition and diagnosis of sepsis had been an essential part of sepsis treatment. According to the surviving sepsis campaign, the early identification and management of sepsis remain unchanged [6].

There is a significant unmet clinical need for a test for the early detection of patients having or developing sepsis. Although the detection and treatment of sepsis are frequently delayed, more rapid administration of sepsis-specific treatments, particularly antibiotics, are associated with improved clinical outcomes, including significantly reduced mortality [7–9]. One of the common causes of sepsis misdiagnosis is the lack of its recognition and contributes to adverse consequences due to delays in definitive antimicrobial treatments [10].

Emergency departments (EDs) have initiated measures to detect sepsis as early as possible, but there still exists the need for a reliable biomarker of sepsis. Existing biomarkers of sepsis such as C-reactive protein (CRP), procalcitonin (PCT), and lactate tests are ordered only if the clinician has already observed a high index of clinical suspicion of sepsis. The average reported accuracy was about 60 to 80% [11–14]. PCT is more useful in guiding antimicrobial therapy than in early sepsis screening [15–17]. Recently, the biomarkers presepsin, interleukin-6 (IL-6), and neutrophil/lymphocyte ratio (N/M ratio) have been investigated. Nonetheless, the performance and the clinical significance of these biomarkers were not better than those of PCT [18–24]. A biomarker with higher sensitivity and negative predictive value (NPV) is mandatory for either early initiation of therapy or early discharge of the patients.

Several studies have suggested that cell population data (CPD) parameters may be potentially useful [25–27]. Neutrophils and monocytes are among the first line to respond to a pathogenic signal generated by the microorganisms. Currently, monocyte distribution width (MDW) is a CE-marked parameter, which can be reported along with complete blood counts (CBCs) from patients presenting to the ED. Crouser et al. showed that MDW has acceptable sensitivity and specificity and is potentially clinically useful for the early detection of patients with or developing sepsis in the ED. They also reported that the diagnostic accuracy further improved by combining MDW with white blood cell (WBC) count [28, 29]. MDW also out-performed the systemic inflammatory response syndrome (SIRS) criteria and the quick SOFA (qSOFA) score [30]. A recent study showed that the performance of MDW was comparable to that of PCT in the infection ward [31]. WBC and differential count are among the most common laboratory tests ordered in patients with suspected infection. It can become a standard tool for the early detection of sepsis in the ED, where most sepsis cases are initially encountered. Despite the results published on these parameters, the performance comparison between MDW and PCT in the emergency department still remains to be assessed.

This study’s primary outcome was to assess the sensitivity and specificity of MDW in differentiating septic and non-septic patients. The cut-off value for MDW was established to maximize sensitivity at an optimal level of specificity. The diagnosis of sepsis based on previous or current definitions was decided by thorough chart review. Then, the performance of MDW and MDW + WBC count was compared to that of PCT. The study results were presented according to the regulation of Standards for Reporting Diagnostic accuracy studies (STARD).

## Methods

### Study design and setting

This was a single-center prospective cohort study. We enrolled patients from the emergency department of a 3000-bed medical center from July to October 2019. The average ED visits were 15,000 patients per month. The study was approved by the Institution Review Board of Chang Gung Memorial Hospital (No. 201900442B0) and registered to the ClinicalTrial.gov (NCT04322942).

### Selection of participants

A research coordinator worked full-time in the ED to screen all non-trauma patients from 8:30 to 17:30, Monday to Friday. The inclusion criteria were: (1) Adult ≥ 20 years of age, (2) Subjects presenting to the ED with the chief complaints of either fever, altered mental status, hypotension, or dyspnea, and (3) Complete blood cell count with differential testing (CBC/DC) was ordered, at presentation, as part of their standard medical care. The exclusion criteria were: (1) Subjects previously enrolled in this study (subjects could not be enrolled more than once in this study), (2) Referred patients who had received antibiotic treatment, (3) Pregnant women, (4) MDW result not reported due to inefficient monocyte count, and (5) Subjects not able to understand or sign informed consent.

### Interventions

No intervention was given to the patients or the caregivers in this study. The decision of blood test, antibiotic treatment, and patient disposition was taken entirely by the physician based on clinical examination. The patients were not enrolled without blood tests. Physicians in-charge were blinded to the MDW result.

### Measurements

After obtaining the informed consent from the patients, their data, including age, gender, height, body weight, vital signs, comorbidities (diabetes mellitus, hypertension, coronary artery disease, chronic obstructive lung disease, asthma, malignancies, congestive heart failure, and chronic kidney disease.), were recorded. DCBUN, creatinine, blood sugar, Na, K, total bilirubin, prothrombin time, lactic acid, and procalcitonin were all measured at the hospital’s central laboratory. We use Roche Elecsys BRAHMS PCT^®^ for the PCT measurement. MDW along with CBC and differential count were measured using Beckman Coulter DxH 900^®^ by the study assistant, according to the manufacturer’s suggestion. The sample was processed and measured within 2 h after collection.

Two separate emergency physicians reviewed the final diagnosis or admission after discharge from the hospital and assigned patients into four groups. The “non-infection” group: patients with no diagnosis related to an infection and did not meet the systemic inflammatory response syndrome (SIRS) criteria; the “infection” group: patients with recognized infectious disease but not meeting SIRS criteria; the “infection + SIRS” group: patients meeting the SIRS criteria with any known infection; the “Sepsis-3” group: patients who met the sepsis-3 criteria of 2017 by which qSOFA score and SOFA score were used accordingly. If the initial assignment did not match, the consensus was made after discussion. Both physicians were blinded to the MDW and procalcitonin data during the process.

### Outcomes

The primary outcome was the diagnostic accuracy of both MDW and PCT presented with the AUC, sensitivity, specificity, positive predictive value (PPV), and negative predictive value (NPV) in predicting infection with/without SIRS and sepsis-3. The secondary outcome was the diagnostic accuracy of WBC count along with MDW in predicting infection and sepsis-3.

### Statistical analysis

Continuous variables were expressed as mean ± SD (standard deviation), and categorical variables were indicated as frequency (%). Statistical significance was determined with Chisq-square test and Kruskal–Wallis test for categorical and continuous variables, respectively. We used boxplots to draw continuous variables. Logistic regression analysis was performed to identify the effect of six variables on the four groups. Diagnostic ability was evaluated in terms of the AUC, sensitivity, specificity, PPV, and NPV along with their 95% CI values. The initial cut-off values were obtained using the Youden index approach, which was optimized iteratively to maximize the sensitivity and specificity. Both WBC count and MDW were dichotomized to 0 and 1 based on their values falling into the normal or abnormal category. WBC count was normal if the recovered value was between 4000 and 11,000/μL. MDW was normal when its recovered value was < 20. Multivariate logistic regression was performed to see if MDW at certain cutoff is an independent predictor of sepsis.

## Results

### Characteristics of study subjects

**Fig. 1:**
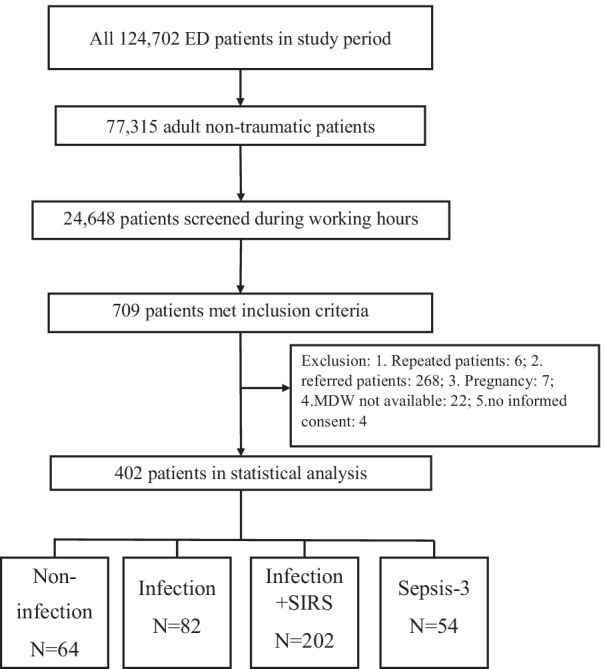
Study enrollment algorithm. 402 patients were enrolled for data analysis from June 2019 to September 2020

From June to September 2020, we screened all the patients admitted to the ED during convenient hours with symptoms suggesting potential sepsis. Among all 124,702 ED patients, 709 met the inclusion criteria, however, 307 patients were excluded because of exclusion criteria. The algorithm is shown in Fig. 1. We enrolled 402 patients for data analysis (Table 1). Two hundred and one patients (50%) were male, and the mean patient age was 63.7 ± 18.9 years. Vital signs at the triage presented with mean were: body temperature 37.7 ± 1.3 °C, respiratory rate (RR) 20.0 ± 4.0/min, and heart rate (HR) 105.0 ± 21.1/min. Systolic and diastolic blood pressure at arrival were 130.3 ± 29.5 and 75.0 ± 17.1 mmHg and 26.4% and 38.1% of patients had diabetes and hypertension, respectively. In addition, 29.6% of the patients had known malignancy. Pneumonia (lower respiratory tract infection) was the most common infection focus (26.7%), followed by urinary tract infection (16.2%), intra-abdominal infection (11.5%), and soft tissue infection (4.2%). WBC count and monocyte distribution width were 11.5 ± 17.9/µL and 22.6 ± 5.3, respectively. The average serum lactate and procalcitonin were 20.3 ± 16.3 mg/dL and 2.5 ± 11.1 ng/mL, respectively. Blood culture was positive in 59 patients (14.7%), and overall mortality was 9.4%.

**Table 1:** Baseline characteristics of the patients

	ALL
Total patient number	402
Age (yrs)	63.7 ± 18.9
Gender, Male N (%)	201 (50)
Body temperature ()	37.7 ± 1.3
Respiratory rate (/min)	20.0 ± 4.0
Heart rate (/min)	105.0 ± 21.1
Systolic blood pressure (mmHg)	130.3 ± 29.5
Diastolic blood pressure (mmHg)	75.0 ± 17.1
White blood cell count (1000/uL)	11.5 ± 17.9
Platelet (1000/uL)	234.7 ± 121.8
Segment (%)	78.1 ± 12.3
Lymphocyte (%)	13.2 ± 9.8
Monocyte (%)	6.7 ± 5.2
Cr (mg/dL)	1.5 ± 1.8
ALT (U/L)	41.4 ± 60.9
Total bilirubin (mg/dL)	1.3 ± 1.1
PT (s)	14.0 ± 3.2
INR	1.3 ± 0.3
aPTT (s)	27.9 ± 6.8
Lactate (mg/dL)	20.3 ± 16.3
MDW	22.6 ± 5.3
Procalcitonin (ng/mL)	2.5 ± 11.1
Comorbidities N (%)	
Diabetes	106 (26.4)
Hypertension	153 (38.1)
Chronic obstructive pulmonary disease	17 (4.2)
Chronic kidney disease	47 (11.7)
Congestive heart failure	13 (3.2)
Malignancy	119 (29.6)
Stroke	25 (6.5)
Liver cirrhosis	14 (3.5)
Infection focus N (%)	
Respiratory tract infection	107 (26.7)
Urinary tract infection	65 (16.2)
Intra-abdominal infection	46 (11.5)
Soft tissue infection	17 (4.2)
Others	166 (41.4)
Identified pathogens	
Gram-positive	17 (4.2)
Gram-negative	205 (51.0)
Bacteremia, N (%)	59 (14.7)
In-hospital mortality, N (%)	35 (9.4)

### Main results

Based on the chart review by two separate emergency physicians blinded to both MDW and PCT results, we assigned the patients to four distinct groups as follows (Table 2), the “non-infection” group (n = 64, 15.9%), the “infection” group (n = 82, 20.4%), the “infection + SIRS” group (n = 202, 50.2%), and the “sepsis-3” group (n = 54, 13.4%).

**Table 2:** Patient characteristics in different groups

	Non-infection	Infection	Infection + SIRS	Sepsis-3	p-value
N (%)	64 (15.9)	82 (20.4)	202 (50.2)	54 (13.4)	
Age (yrs)	60.1 ± 17.8	62.3 ± 20.2	62.8 ± 18.9	73.2 ± 15.7	0.001
Gender, Male N (%)	29 (45.3)	38 (46.3)	103 (51.0)	31 (57.4)	0.5191
Body temperature ()	37 ± 1.2	37.2 ± 0.9	38.2 ± 1.2	37.6 ± 1.5	< 0.0001
Respiratory rate (/min)	20 ± 3.7	17.9 ± 2.3	19.7 ± 3.6	24.6 ± 4.6	< 0.0001
Heart rate (/min)	96.6 ± 22.9	90.0 ± 15.4	112.4 ± 17.3	109.6 ± 24.2	< 0.0001
Systolic blood pressure (mmHg)	130.5 ± 29.5	128 ± 30.0	135.3 ± 27.6	114.8 ± 30.7	0.0005
Diastolic blood pressure (mmHg)	76.3 ± 18.8	75.5 ± 16.6	76.3 ± 15.7	68.1 ± 19.5	0.0046
White blood cell count (1000/uL)	8.8 ± 5.0	7.9 ± 3.3	12.1 ± 6.0	18.3 ± 46.4	< 0.0001
Platelet (1000/uL)	250.9 ± 113.1	229.2 ± 102.4	237.5 ± 127.4	214.6 ± 130.9	0.2594
Segment (%)	74.0 ± 9.9	74.0 ± 12.4	80.4 ± 11.7	80.3 ± 14.1	< 0.0001
Lymphocyte (%)	17.4 ± 8.9	16.6 ± 9.2	11.0 ± 8.9	11.2 ± 11.7	< 0.0001
Monocyte (%)	6.5 ± 2.9	7.0 ± 5.1	7.1 ± 6.2	5.5 ± 3.1	0.142
Cr (mg/dL)	1.7 ± 2.0	1.5 ± 1.7	1.4 ± 1.7	1.8 ± 1.7	0.1564
Total bilirubin (mg/dL)	1.4 ± 1.5	0.9 ± 0.6	1.2 ± 1.0	1.7 ± 1.7	0.2965
INR	1.3 ± 0.3	1.2 ± 0.1	1.3 ± 0.3	1.5 ± 0.3	0.0003
MDW	18.5 ± 3.3	20.8 ± 4.0	23.5 ± 4.6	26.8 ± 7.0	< 0.0001
Procalcitonin (ng/mL)	0.2 ± 0.3	0.4 ± 1.5	1.9 ± 7.9	10.3 ± 24.6	< 0.0001
Bacteremia, N (%)	1 (1.6)	3 (3.7)	35 (17.3)	20 (37.0)	< 0.0001
In-hospital mortality, N (%)	1 (1.9)	2 (2.6)	13 (6.8)	19 (36.5)	< 0.0001

**Fig. 2:**
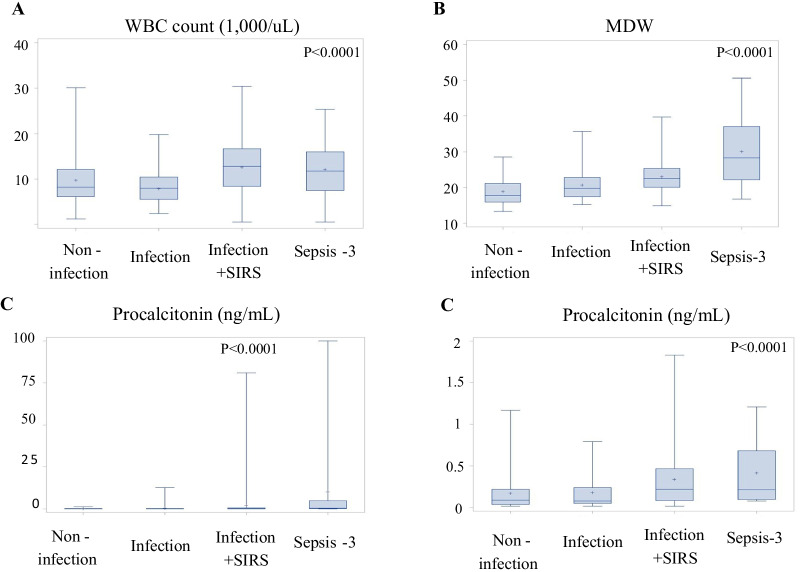
Main laboratory results measured in each group. There was no significant difference in WBC count between four groups (**A**). MDW (**B**) and procalcitonin (**C**) measurement increased in patients with infection. Patient met sepsis-3 criteria had the highest MDW and PCT level. **D** The boxplot which the extreme outliers were removed

Among the four groups, age and gender distribution were similar. Patients in the infection + SIRS group had the highest initial body temperature (38.2 ± 1.2 ℃, p < 0.001). Patients in the sepsis-3 group had a higher respiratory rate (24.6 ± 4.6/min) and lower systolic blood pressure (114.8 ± 30.66 mmHg, p < 0.001). There was no difference regarding the underlying condition of the patients. Laboratory examinations, including BUN, sodium, potassium, total bilirubin, alanine aminotransferase, were not different between groups. Infection + SIRS and Sepsis-3 groups had higher serum creatinine and INR (p < 0.05). WBC count in each group was 8.8 ± 5.0, 7.9 ± 3.3, 12.1 ± 6.0, and 18.3 ± 46.4 1000/uL, respectively (Fig. 2A, p < 0.05). MDW in each group was measured to be 18.5 ± 3.3, 20.8 ± 4.0, 23.5 ± 4.6, and 26.8 ± 7.0, respectively (Fig. 2B, p < 0.05). The estimated PCT in each group was 0.2 ± 0.3, 0.4 ± 1.5, 1.9 ± 7.9, and 10.3 ± 24.6 ng/mL, respectively (Fig. 2C, p < 0.05). Additional file 2: Table S1 reported continuous variables in median.

**Fig. 3:**
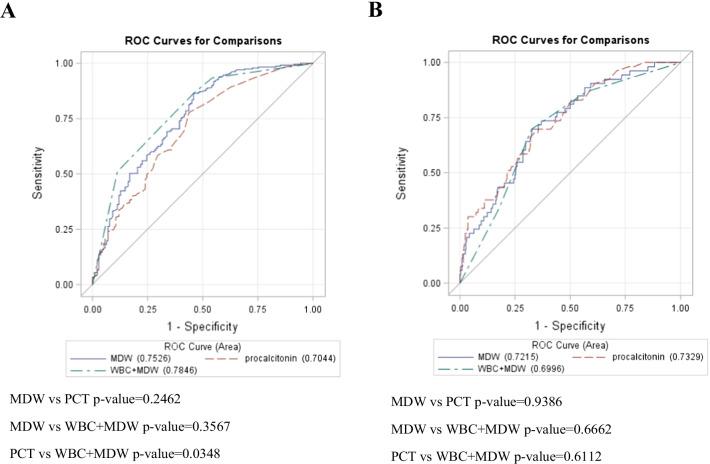
Receiver operating characteristic (ROC) curve analysis of MDW and PCT in predicting infection + SIRS (**A**) and sepsis-3 (**B**)

Next, we evaluated the diagnostic accuracy of MDW, PCT and MDW + WBC with AUC analysis. For predicting infection + SIRS, the AUC of MDW and PCT was 0.753 (0.701–0.804) and 0.704 (0.65–0.759), respectively (Table 3 and Fig. 3A). The best MDW cut-off values were estimated to be 19.3, which is similar to the suggested cut-off in previous studies. Based on the earlier reports and our data, we defined normal WBC count in the range of 4000 to 11,000/µL [28]. When normal WBC count and normal MDW were used as the cut-off, the AUC predicting infection + SIRS and sepsis-3 was 0.784 (0.738–0.829), and 0.699 (0.631–0.768), respectively. The sensitivity, specificity, PPV, and NPV of MDW using 20 as the cutoff in predicting infection + SIRS were 86.4%, 54.2%, 76.4%, and 70%, compared to 32.9%, 88%, 82.5%, and 43.4% using 0.5 ng/mL as the PCT cutoff value. On combing MDW and WBC count, the sensitivity and NPV further increased to 93.4% and 80.3%, respectively. In predicting sepsis-3, the AUC of MDW and PCT was 0.722 (0.652–0.792), and 0.733 (0.663–0.802), with the best MDW cut-off value of 23.4 and 0.31 ng/mL (Fig. 3B). When the WBC count was added to the model, the AUC was 0.700 (0.631–0.768). MDW, using 20 as cutoff, exhibited sensitivity, specificity, PPV, and NPV of 90.6%, 37.1%, 18.7%, and 96.1%, respectively, compared to 49.1%, 78.6%, 26.8%, and 90.6% when 0.5 ng/mL PCT was used as cutoff. When WBC count was combined with MDW, the sensitivity and NPV were 86.8% and 95.3%, respectively. Multivariate logistic regression showed that MDW > 20 is an independent predictor of both infection + SIRS and sepsis-3. The odds ratio is 3.991 (2.152–7.402) and 6.472 (2.195–19.09) (Additional file 2: Table S2), respectively.

**Table 3:** Diagnostic accuracy of MDW and PCT in predicting infection + SIRS and sepsis-3

	AUC	95% C.I	Cut-off	Sensitivity (%)	Specificity (%)		PPV (%)		NPV (%)
Infection + SIRS									
MDW	0.753	(0.701–0.804)	19.26	86.4 (80.4–89.5)	54.2 (44.7–61.2)		76.4 (70.1–80.4)		70.0 (58.1–76.5)
Procalcitonin	0.704	(0.650–0.759)	0.10	77.8 (65.9–78.8)	56.3 (49.2–66.0)		75.3 (68.4–80.0)		59.7 (46.3–62.8)
WBC_N + MDW_N	0.784	(0.738–0.829)	–	93.4 (90.0–96.3)	45.8 (36.8–53.3)		74.7 (69.1–79.0)		80.3 (70.6–88.6)
MDW			20.00	80.7 (73.9–84.3)	56.3 (47.4–63.8)		76.0 (69.8–80.3)		63.0 (52.7–69.7)
Procalcitonin			0.50	32.9 (26.7–38.8)	88.0 (81.5–92.9)		82.5 (73.2–89.3)		43.4 (37.5–49.2)
Sepsis-3									
MDW	0.722	(0.652–0.792)	23.41	69.8 (56.4–82.0)		67.5 (63.2–73.2)		25.5 (18.9–33.5)	93.3 (90.0–96.4)
Procalcitonin	0.733	(0.664–0.802)	0.31	67.9 (51.7–78.5)		66.6 (62.8–73.1)		24.5 (17.9–32.8)	92.9 (88.6–95.6)
WBC_N + MDW_N	0.700	(0.631–0.768)	–	86.8 (75.7–94.6)		42.8 (37.6–48.2)		19.5 (14.4–24.6)	95.3 (91.0–98.2)
MDW			20.00	90.5 (79.7–96.9)		37.1 (32.3–42.7)		18.7 (13.9–23.5)	96.1 (91.6–98.8)
Procalcitonin			0.50	49.0 (33.7–60.6)		78.6 (73.8–82.9)		26.8 (17.6–36.0)	90.6 (86.3–93.5)

*AUC* Area under receiver operating characteristic (ROC) curve, *PPV* positive predictive value, *NPV* negative predictive value

## Discussion

In 2017, sepsis was re-defined as life-threatening organ dysfunction caused by an overwhelming host immune response. If the patient meets the criteria of sepsis-3, the mortality remains as high as greater than 30%. Delayed recognition and treatment of sepsis can result in worse patient outcomes. The new definition focused on the identification of the patients with the worst prognosis. However, it took thorough laboratory tests, including liver function, renal function, and arterial blood gas analysis, to complete the SOFA score. Some studies have suggested that it is not feasible for an emergency physician to recognize or exclude patients with the potential risk of sepsis and decide patient disposition [32–34]. This study showed that MDW only or combined with WBC count can be used as a readily available biomarker in the ED to predict bacterial infection.

To date, most clinicians utilize CRP and PCT to predict sepsis in the ED. The performance of PCT was better than that of CRP in earlier studies [14, 20, 35]. As an early screening tool for sepsis, its sensitivity and specificity were around 80% and 75%, respectively. Nonetheless, the accuracy of PCT in predicting patients fulfilling sepsis-3 criteria remains low. PCT is generally used to exclude severe sepsis because of its high NPV. The attempt to develop a more accurate diagnostic modality had never stopped. Presepsin, interleukin-6, pentraxin-3, and neutrophil/lymphocyte ratio were some of the biomarkers developed and investigated recently [24, 36, 37]. Nevertheless, PCT remains by far the most common biomarker of sepsis. MDW is different from most of the biomarkers mentioned because it can be estimated as a part of the CBC count measurement without extra-expanse, at least in the health care system of Taiwan. It could be advantageous if the diagnostic accuracy is comparable to or even better than the biomarkers we are currently using.

Fever is the most common presenting symptom of infection. Nonetheless, patients with sepsis may present to the ED with a wide variety of clinical signs, including malaise, shortness of breath, conscious disturbance, hypotension, etc. [5]. Early detection depends on clinical suspicion and the test results of various biomarkers. We intended to design a study that is more relevant to the clinical setting. Thus, the study excluded patients who did not undergo any blood test according to clinical judgment. The setting was similar to the actual practice in the ED. The mortality of patients fulfilling sepsis-3 was also similar to that mentioned in the previous report. Fever was not found to be a good indicator of severe sepsis. Sepsis-3 criteria predicted in-hospital mortality was better than previous sepsis criteria in our patient group. The mortality rate was also similar to that mentioned in the previous report.

Sepsis-3 focused on those patients with the worst outcome. Even so, in most cases, emergency physicians had to decide when to discharge with acceptable risk. Biomarkers with higher sensitivity helped us to initiate the sepsis treatment protocol as early as possible. Previous sepsis criteria were more feasible for early screening. The accuracy of MDW alone or MDW and WBC count in predicting infection + SIRS were both better than that of PCT. The best cut-off value of MDW was 19.26, which was similar to 20 in the previous report. Besides, MDW also provided better sensitivity than PCT at 0.5 ng/mL cut-off value. MDW, in this case, was a better biomarker than PCT for early sepsis screening.

In terms of predicting sepsis-3, both MDW and PCT had comparable AUC. However, using 20 as the cut-off value, MDW provided significantly higher sensitivity and NPV than those by PCT at 0.5 ng/mL cut-off value. The accuracy further increased while WBC count was added. PCT was observed to be a specific but not a sensitive biomarker, especially in predicting sepsis-3. It is challenging to exclude critical patients using PCT in the clinical setting. Serial PCT follow-up is another way to predict patient outcomes [38, 39]. MDW, at the cut-off value of 20, provided an excellent negative predictive value to exclude sepsis-3. Overall, MDW had better performance in both early screening of sepsis and patient outcome prediction. WBC count had relatively good sensitivity but low specificity traditionally [6]. Combining MDW and WBC count increased both sensitivity and NPV in predicting infection + SIRS but not in sepsis-3. Nonetheless, the specificity decreased accordingly. Clinicians must interpretate the result with caution to minimize unnecessary antibiotic usage or admission.

Monocytes, including macrophages and dendritic cells, serve as the first-line responder of innate and adaptive immunity. Microorganisms activate the immune system through various pattern recognition receptors on monocytes. MDW can measure the size and shape change of monocytes during the activation and differentiation process. This makes MDW a unique and novel biomarker compared to other protein-based markers. It takes hours for the target cells to start protein production to complete the transcription and translation processes. The differentiation of monocytes in the circulation begins relatively early in the sepsis cascade, which could be one reason for the increased sensitivity of MDW in the sepsis diagnosis.

One of the limitations of MDW is that the value is not available in patients with a monocyte event < 100 count in the peripheral blood sample. The left-shift of WBC subtypes is one of the earliest used biomarkers in the sepsis diagnosis. In this study, we assumed that the low monocyte percentage was due to the WBC left-shift. That is, patients with low monocyte count might have a higher chance of sepsis. In our study, MDW was not measurable in 22 patients, and 20 (91%) and 10 (45%) patients were in the infection + SIRS and sepsis-3 group, respectively. Although the possibility of sepsis was higher in these patients, we suggested the physicians use other sepsis biomarkers if the MDW value were not available.

In most health care systems, PCT is a relatively expensive biomarker. It is thrice as costly as CBC/DC or CRP in Taiwan’s National Health Insurance program. An emergency physician can not order PCT in all suspected patients for sepsis screening. MDW, as part of the CBC/DC report, could be an economical yet accurate screening tool in the ED. PCT can be used as the second-line sepsis biomarker if the diagnosis is still doubtful.

Our results should be interpreted in the context of some limitations. First, this study is a single-center study in only one ED. The results might not be generalizable to all EDs. We might need a multi-center study to validate the results. Second, we enrolled patients only during the working hours. There might be potential selection bias. Third, we enrolled patients with specific symptoms instead of consecutive patients in the ED and excluded those without laboratory testing. Nonetheless, we believe that it best fits the ED practice model. Fourth, PCT might be falsely positive in patients with malignancy [40, 41]. We found that the performance of PCT diminished in patients with malignancy (Additional file 1: Fig. S1). However, further study is mandated to address this issue. Lastly, most of the documented pathogens are Gram-negative bacteria. Lipopolysaccharide (LPS) is one of the most important bacterial components involved in monocyte activation [42]. The role of MDW in Gram-positive or fungal infection needs further study in the future.

## Conclusions

Early recognition of septic patients remains challenging. Clinical suspicion remains even in the era of various biomarkers and artificial intelligence. Biomarkers help physicians to decide the patient's disposition confidently. Our results showed that MDW increased among patients with infection by severity. In conclusion, MDW is a more sensitive biomarker than PCT in predicting infection + SIRS or sepsis-3 in the ED. The suggested cut-off was 20. MDW < 20 shows a higher NPV to exclude sepsis-3. Thus, it could be a useful screening tool for sepsis detection in the ED.

## Supplementary Information

**Additional file 1: Figure S1.** The performance of MDW and PCT in patients with underlying malignancy. The ROC curve of MDW and PCT predicting infection+sepsis in patients without (A) and with (B) malignancy. AUC of PCT predicting sepsis is lower but not statistically significant. The ROC curve of MDW and PCT predicting sepsis-3 in patients without (C) and with (D) malignancy. There is no significant difference.

**Additional file 2: Table S1.** Patient characteristics in different groups (continuous variables in median). **Table S2.** Multivariate logistic regression model to predict infection+SIRS and sepsis-3.

## Data Availability

The datasets used and/or analysed during the current study are available from the corresponding author on reasonable request.

## Abbreviations

AUC: Area under curve
CBC: Complete blood count
CPD: Cell population data
CRP: C-reactive protein
ED: Emergency department
IL-6: Interleukin-6
MDW: Monocyte distribution width
NPV: Negative predictive value
PCT: Procalcitonin
PPV: Positive predictive value
SD: Standard deviation
SIRS: Systemic inflammatory response syndrome
