# Supplementary material for: Comparison of the diagnostic accuracy of monocyte distribution width and procalcitonin in sepsis cases in the emergency department: a prospective cohort study
Source: BMC Infect Dis. 2022 Jan 4;22:26. doi: 10.1186/s12879-021-06999-4 (PMC8725440; doi:10.1186/s12879-021-06999-4)
Supplement: Supplementary file 1 — Additional file 1: Figure S1. The performance of MDW and PCT in patients with underlying malignancy. The ROC curve of MDW and PCT predicting infection+sepsis in patients without (A) and with (B) malignancy. AUC of PCT predicting sepsis is lower but not statistically significant. The ROC curve of MDW and PCT predicting sepsis-3 in patients without (C) and with (D) malignancy. There is no significant difference. [file 12879_2021_6999_MOESM1_ESM.docx]

**A. No Malignancy**

**B. Malignancy**


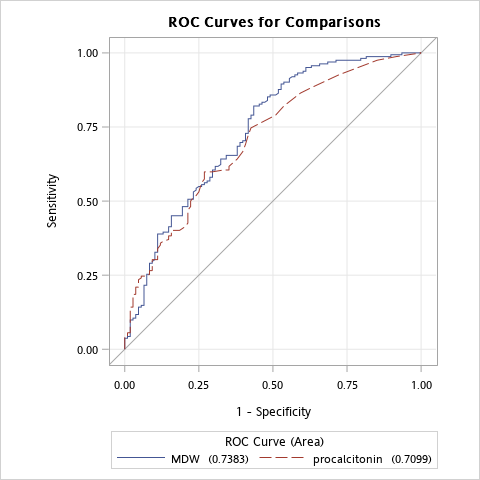

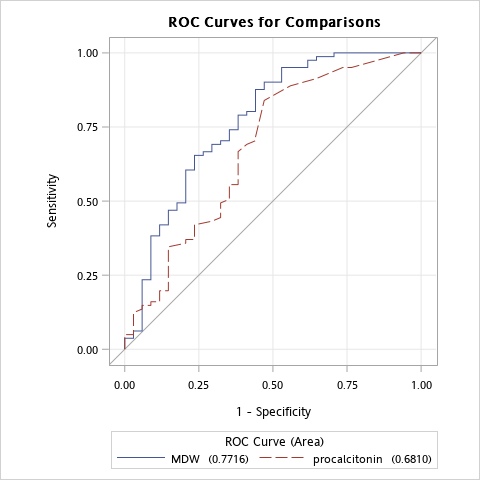


MDW vs procalicitonin p-value=0.2238

MDW vs procalicitonin p-value=0.6549


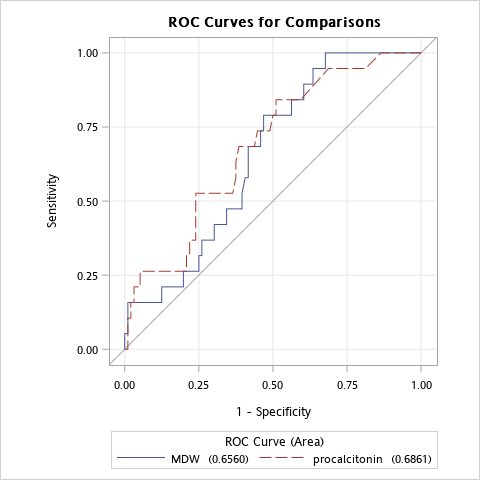

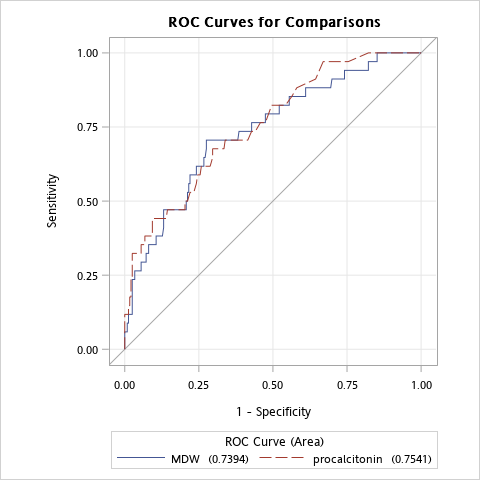


MDW vs procalicitonin p-value=0.7576

MDW vs procalicitonin p-value=0.9368

**D. Malignancy**

**C. Non Malignancy**
