# Supplementary material for: Comparison of the diagnostic accuracy of monocyte distribution width and procalcitonin in sepsis cases in the emergency department: a prospective cohort study
Source: BMC Infect Dis. 2022 Jan 4;22:26. doi: 10.1186/s12879-021-06999-4 (PMC8725440; doi:10.1186/s12879-021-06999-4)
Supplement: Supplementary file 2 — Additional file 2: Table S1. Patient characteristics in different groups (continuous variables in median). Table S2. Multivariate logistic regression model to predict infection+SIRS and sepsis-3. [file 12879_2021_6999_MOESM2_ESM.docx]

**Table S1. Patient characteristics in different groups (continuous variables in median)**

|  | Non-infection | Infection | Infection+SIRS | Sepsis-3 | p-value |
| --- | --- | --- | --- | --- | --- |
| N (%) | 64 (15.9) | 82 (20.4) | 202 (50.2) | 54 (13.4) |  |
| Age (yrs) | 62 | 66 | 64.5 | 76 | 0.001 |
| Gender, Male N(%) | 29(45.31) | 38(46.34) | 103(50.99) | 31(57.41) | 0.5191 |
| Body temperature () | 37.05 | 37.45 | 38.3 | 37.85 | <0.0001 |
| Respiratory rate (/min) | 19 | 18 | 19 | 24 | <0.0001 |
| Heart rate (/min) | 95 | 87 | 111.5 | 107.5 | <0.0001 |
| Systolic blood pressure (mmHg) | 134 | 125.5 | 133 | 116 | 0.0005 |
| Diastolic blood pressure (mmHg) | 75.5 | 72.5 | 75.5 | 62.5 | 0.0046 |
| White blood cell count (1,000/uL) | 7.7 | 7.8 | 12.1 | 11.45 | <0.0001 |
| Platelet (1,000/uL) | 229 | 219 | 222 | 197 | 0.2594 |
| Segment (%) | 74.25 | 76.05 | 82.95 | 83 | <0.0001 |
| Lymphocyte (%) | 15.55 | 15 | 8.5 | 8 | <0.0001 |
| Monocyte (%) | 5.6 | 6.1 | 6 | 5.05 | 0.142 |
| Cr (mg/dL) | 0.96 | 0.98 | 0.98 | 1.23 | 0.1564 |
| Total bilirubin (mg/dL) | 0.8 | 0.8 | 1 | 1 | 0.2965 |
| INR | 1.25 | 1.1 | 1.2 | 1.3 | 0.0003 |
| MDW | 17.66 | 20.88 | 22.6 | 24.51 | <0.0001 |
| Procalcitonin (ng/mL) | 0.08 | 0.09 | 0.23 | 0.49 | <0.0001 |
| Bacteremia, N(%) | 1(1.56) | 3(3.7) | 35(17.33) | 20(37.04) | <0.0001 |
| In-hospital mortality, N(%) | 1(1.85) | 2(2.63) | 13(6.81) | 19(36.54) | <0.0001 |

**Table S2. Multivariate logistic regression model to predict infection+SIRS and sepsis-3**

**Infection+SIRS**

| Variable | β | Odds Ratio | 95% Confidence Interval | p-value |
| --- | --- | --- | --- | --- |
| Intercept | 1.027 |  |  |  |
| MDW>20 | 0.692 | 3.991 | (2.152,7.402) | <0.001 |
| AGE>60 | 0.548 | 2.991 | (1.523,5.872) | 0.002 |
| BT>38 | 1.037 | 7.956 | (4.057,15.60) | <0.001 |
| RR>20 | 0.536 | 2.920 | (1.477,5.774) | 0.002 |
| HR>104.03 | 0.881 | 5.827 | (3.087,11.00) | <0.001 |
| Lymphocyte>9.1 | -0.585 | 0.310 | (0.167,0.579) | <0.001 |

**Sepsis-3**

| Variable | β | Odds Ratio | 95% Confidence Interval | p-value |
| --- | --- | --- | --- | --- |
| Intercept | -1.633 |  |  |  |
| MDW>20 | 0.934 | 6.472 | (2.195,19.09) | 0.001 |
| RR>21 | 1.521 | 20.95 | (8.971,48.94) | <0.001 |
| SBP>97.07 | -1.256 | 0.081 | (0.031,0.211) | <0.001 |
